# Supplementary material for: Development of a clinical predictive score for allergic reactions during oral food challenges in pediatric patients
Source: PLoS One. 2025 Apr 24;20(4):e0322152. doi: 10.1371/journal.pone.0322152 (PMC12021193; doi:10.1371/journal.pone.0322152)
Supplement: S1 Table — (DOCX) [file pone.0322152.s001.docx]

| Culprit food tested at OFCs | Number of patients (N) | Patients with allergic reactions during OFCs | |
| --- | --- | --- | --- |
|  |  | N | Percent |
| Cow milk | 76 | 5 | 6.6 |
| Egg yolk | 22 | 1 | 4.5 |
| Egg white | 19 | 4 | 21.0 |
| Seafood | 19 | 3 | 15.8 |
| Peanut | 6 | 1 | 16.7 |
| Other | 36 | 8 | 22.2 |

Supplement 1 Culprit foods of the study population undergoing OFCs
