# Supplementary material for: Development of a clinical predictive score for allergic reactions during oral food challenges in pediatric patients
Source: PLoS One. 2025 Apr 24;20(4):e0322152. doi: 10.1371/journal.pone.0322152 (PMC12021193; doi:10.1371/journal.pone.0322152)
Supplement: S2 Table — (DOCX) [file pone.0322152.s002.docx]

Supplement 2 The allergic reaction rate during OFCs for each score

| Score | Number of patients (N) | Having  allergic reactions | |
| --- | --- | --- | --- |
|  |  | N | (%) |
| 0 | 53 | 4 | 7.5 |
| 1 | 82 | 5 | 6.1 |
| 2 | 35 | 9 | 25.7 |
| 3 | 9 | 4 | 44.4 |
